# Antiproliferative and apoptosis-inducing activity of schisandrin B against human glioma cells

**DOI:** 10.1186/s12935-015-0160-x

**Published:** 2015-02-04

**Authors:** Qun Li, Xiang-he Lu, Cheng-de Wang, Lin Cai, Jiang-long Lu, Jin-sen Wu, Qi-chuan Zhuge, Wei-ming Zheng, Zhi-peng Su

**Affiliations:** Department of Neurosurgery, The First Affiliated Hospital of Wenzhou Medical University, Wenzhou, 325000 China

**Keywords:** Glioma cells, Schisandrin B, Proliferation, Cell cycle, Apoptosis

## Abstract

**Background:**

Malignant glioma is the most devastating and aggressive tumour in the brain and is characterised by high morbidity, high mortality and extremely poor prognosis. The main purpose of the present study was to investigate the effects of schisandrin B (Sch B) on glioma cells both *in vitro* and *in vivo* and to explore the possible anticancer mechanism underlying Sch B-induced apoptosis and cell cycle arrest.

**Methods:**

The anti-proliferative ability of Sch B on glioma cells were assessed by MTT and clony formation assays. Flow cytometric analysis was used to detect cell cycle changes. Apoptosis was determined by Hoechst 33342 staining and annexin V/PI double-staining assays. The mitochondrial membrane potential was detected by Rhodamine 123 staining. The *in vivo* efficacy of Sch B was measured using a U87 xenograft model in nude mice. The expressions of the apoptosis-related and cell cycle-related proteins were analysed by western blot. Student’s t-test was used to compare differences between treated groups and their controls.

**Results:**

We found that Sch B inhibited growth in a dose- and time-dependent manner as assessed by MTT assay. In U87 and U251 cells, the number of clones was strongly suppressed by Sch B. Flow cytometric analysis revealed that Sch B induced cell cycle arrest in glioma cells at the G0/G1 phase. In addition, Sch B induced glioma cell apoptosis and reduced mitochondrial membrane potential (ΔΨm) in a dose-dependent manner. Mechanically, western blot analysis indicated that Sch B induced apoptosis by caspase-3, caspase-9, PARP, and Bcl-2 activation. Moreover, Sch B significantly inhibited tumour growth *in vivo* following the subcutaneous inoculation of U87 cells in athymic nude mice.

**Coclusions:**

In summary, Sch B can reduce cell proliferation and induce apoptosis in glioma cells and has potential as a novel anti-tumour therapy to treat gliomas.

## Background

Glioma, which is characterised by rapid proliferation and diffuse invasion, is one of the most common malignant brain tumours in adults. In the United States, malignant gliomas account for approximately 70% of malignant primary brain tumours in adults [1]. Because surgical treatment is unable to remove all malignant glioma cells, it is difficult to eliminate this type of malignancy. As a result, malignant gliomas are associated with high morbidity, high mortality and extremely poor prognosis [2-7]. Tumour recurrence usually recurs within 6.9 months, resulting in a median patient survival of just 12–15 months following diagnosis [1]. Moreover, high drug resistance and the blood–brain barrier usually lower the efficacy of chemotherapy drugs [8]. Hence, new effective treatments and drugs are urgently needed to improve outcomes for patients with malignant gliomas.

**Figure 1:**
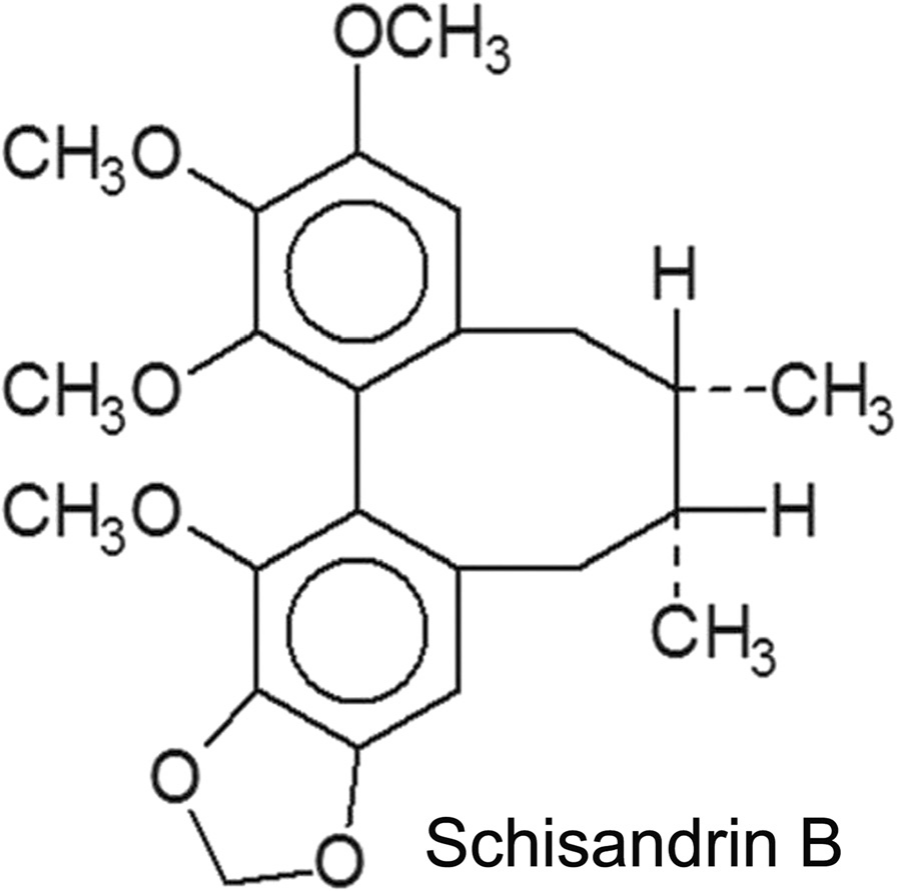
**The chemical structure of Sch B.**

Schisandrin B (Sch B, Figure 1), extracted from the fruit of *Schisandra chinensis* Baill, has been used in traditional Chinese medicine to treat hepatitis and myocardial disorders [9]. In addition, Sch B has been reported to possess antitumor activity in various human cancers, including breast cancer, gastric cancer and hepatoma [10-12]. Previous studies have shown that Sch B can enhance the doxorubicin-induced apoptosis of human breast cancer cells but not normal cells via caspase-9 activation [13]. Sch B attenuates cancer invasion and metastasis [14] and inhibits ATR protein kinase activity when DNA damage occurs [15]. However, to the best of our knowledge, the effects of Sch B on glioma cells and the underlying mechanisms of these effects have not been previously reported. In this study, we investigated the effects of Sch B on the proliferation and apoptosis of human glioma cell lines (including the U87 and U251 cell lines) and the possible molecular mechanisms underlying these actions, which indicate that Sch B may be a new natural anti-tumour medicine for gliomas.

## Results

### Sch B inhibits the proliferation and viability of glioma cells in a dose-dependent manner

**Figure 2:**
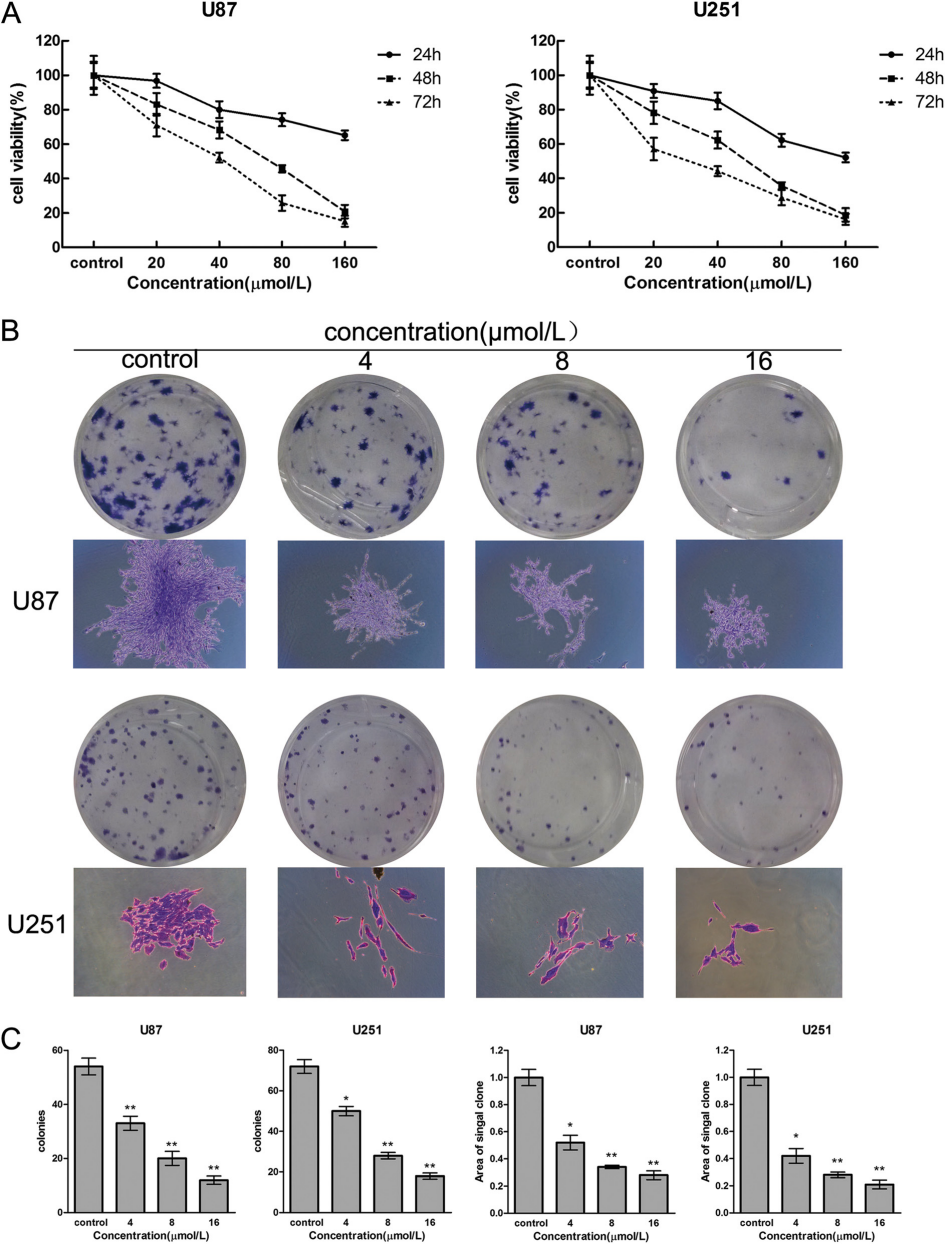
**Sch B reduces the proliferation and viability of glioma cells. A**. U87 and U251 cells were treated with various concentrations of Sch B for 24, 48 and 72 h. Cell viability and IC_50_ values were measured using the MTT assay. **B** and **C**. U87 and U251 cells were treated with Sch B(4, 8, 16μmol/L ) for 48 h and were then allowed to form colonies in fresh medium for 14 days. Representative Giemsa staining pictures of the colonies were shown **(B)**. The numbers of colonies of U87 and U251 cells **(C)** were counted. The data shown are the mean ± SD of three independent experiments. *P < 0.05; **P < 0.01; ***P < 0.001 vs. the control group.

We investigated the effect of Sch B on the proliferation of various glioma cells using the MTT assay. U87 and U251 cells were treated with various concentrations (0, 20, 40, 80 and 160 μmol/L for both cell lines) of Sch B for 24, 48, and 72 h, then their viability was evaluated. As shown in Figure 2A, Sch B induced a dose- and a time-dependent reduction in the viability of U87 and U251 cells. The IC50 values (the drug concentration that inhibited 50% of the cells) of Sch B in U87 and U251 cells at 48 h were approximately 70 μmol/L and 60 μmol/L, respectively. We next tested the effect of Sch B on the ability of U87 and U251 cells to form colonies. Sch B induced a dose-dependent decrease in colony formation in both glioma cell lines (Figure 2B). Moreover, statistical analysis confirmed that the mean size of the control colonies was larger than that of the Sch B-treated group (Figure 2B). These data support the ability of Sch B to significantly inhibit the proliferation of glioma cells.

### Sch B induces G0/G1 phase arrest by regulating the expression of cell cycle-related proteins in U87 and U251 cells

**Figure 3:**
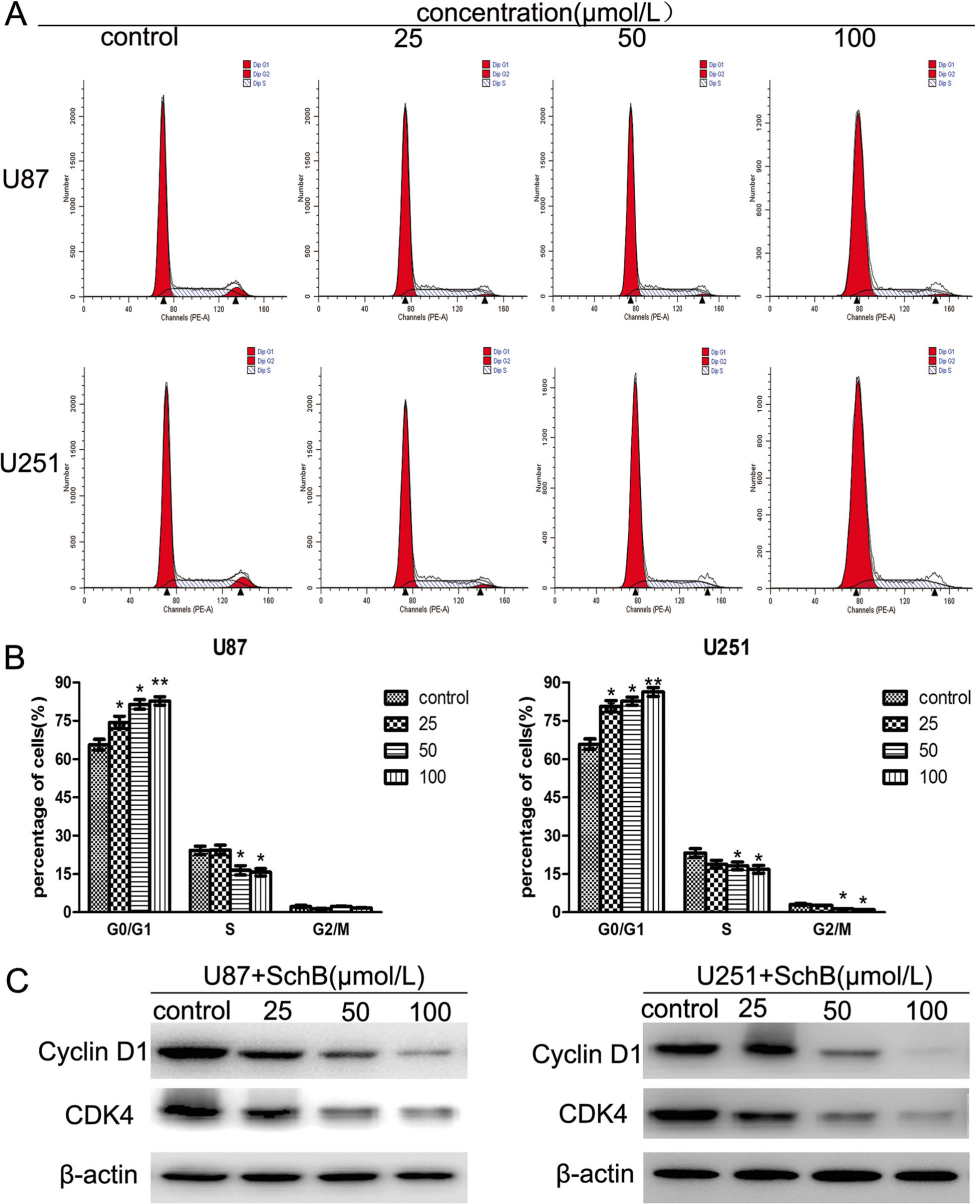
**Effect of Sch B on cell cycle progression in glioma cells. A**. U87 and U251 cells were exposed to various concentrations of Sch B (0, 25, 50, 100 μmol/L) for 48 h and then analysed using flow cytometry. **B**. The percentages of the total cell population in the G0/G1, S, and G2/M phases of the cell cycle are calculated. **C**. Effects of Sch B on the protein levels of cyclin D1 and CDK4 in U87 and U251 cells were measured by western blot analysis, with β-actin as a loading control.

To further understand the mechanism underlying the Sch B-mediated growth-inhibitory effect in human glioma cells, we performed cell cycle analysis in U87 and U251 cells in the presence of Sch B. Sch B-mediated changes in the cell cycle of U87 and U251 cells are shown in Figure 3A and B. After treatment with Sch B for 48 h, the percentage of G0/G1-phase cells dramatically increased (from 63.2% to 82.5% for U87 cells, p < 0.01; from 64.5% to 84.7% for U251 cells, p < 0.01), whereas the percentage of cells in S-phase slightly decreased compared with that of control cells.

Next, we assessed the levels of the G0/G1 phase-related proteins cyclin D1 and cyclin-dependent kinase 4 (CDK4) using western blot analysis. As shown in Figure 3C, Sch B treatment reduced the expression of cyclin D1 and CDK4 in a dose-dependent manner, which is consistent with G0/G1 phase arrest induced by Sch B.

### Sch B induces apoptosis in human glioma cells

**Figure 4:**
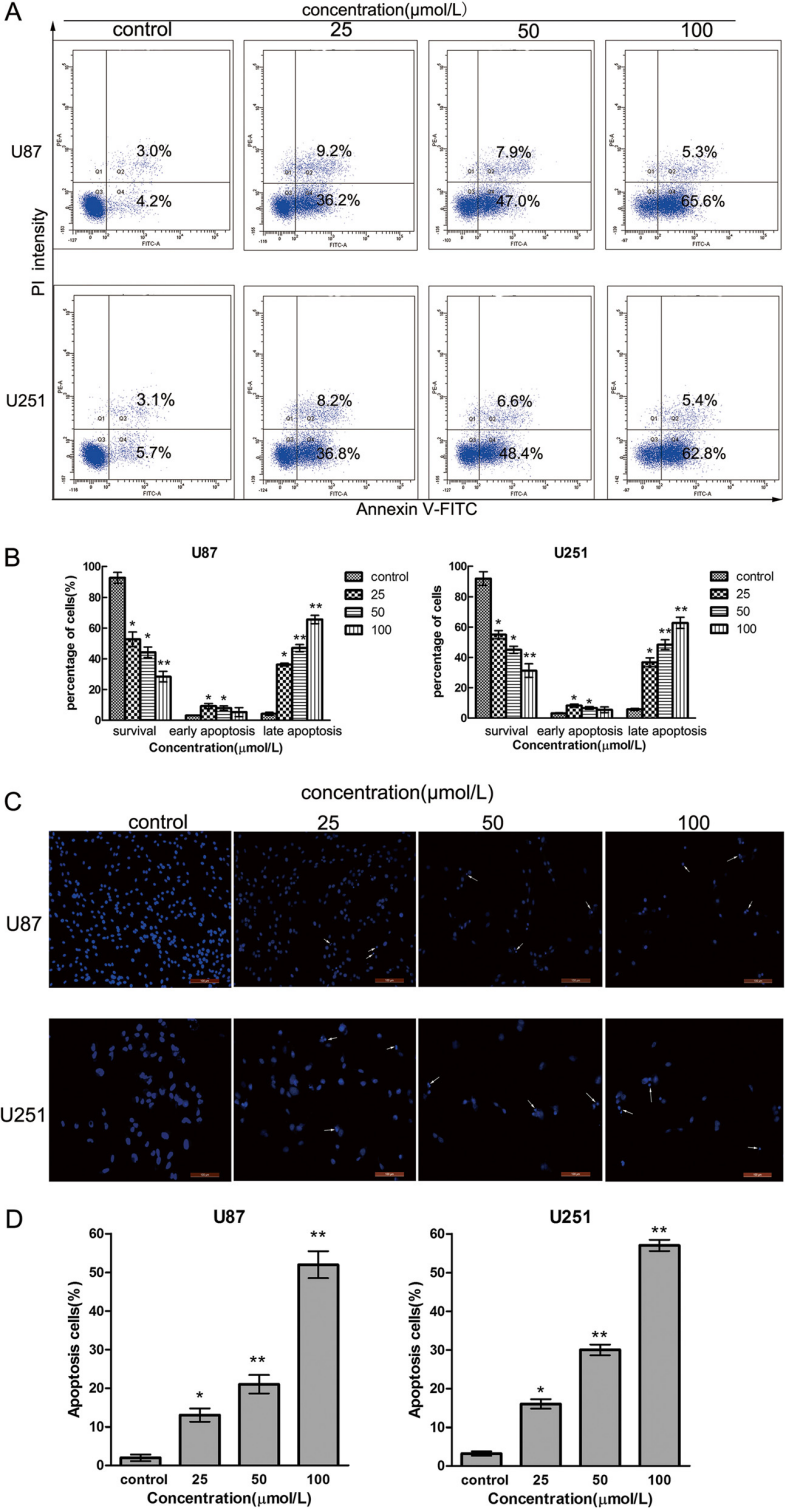
**Sch B induces apoptosis in U87 and U251 cells.** U87 and U251 cells were exposed to various concentrations of Sch B for 48 h. **A**. Sch B treated cells were stained with annexin-V/propidium iodide (PI). The Q3 (annexin-V−/PI−), Q4 (annexin-V+/PI−) and Q2 (annexin-V+/PI+) quadrants represent the populations of normal, early apoptotic and late apoptotic cells, respectively. **B**. Percentages of surviving cells and early and late apoptotic cells are shown as the means ± SD (n = 3). **C**. Apoptotic nuclear morphological changes induced by Sch B (0, 25, 50, 100 μmol/L) for 48 h were observed using Hoechst 33342 staining in U87 and U251 cells. The arrowheads indicate apoptotic cells, which exhibited highly condensed and fragmented nuclear morphologies. **D**. Percentages of apoptotic cells are shown as the means ± SD (n = 3). *P < 0.05, **P < 0.01, ***P < 0.001 compared with the control group.

To further examine whether Sch B restrained the growth of U87 and U251 cells through the induction of apoptosis, we performed annexin V/propidium iodide (PI) double staining followed by flow cytometric analysis after treating the cells with various doses of Sch B. One of the distinct phenomenon of apoptosis is the translocation of phosphatidylserine (PS) from the inner leaflet to the outer leaflet of the plasma membrane. As the human anticoagulant annexin-V has a high affinity for PS, apoptotic cells can be identified via fluorophore-labelled annexin-V. In addition, PI can detect necrotic cells due to its ability to enter disrupted cell membranes. Therefore, various cell populations can be easily distinguished using annexin V/PI staining. As shown in Figure 3A,B, The number of late apoptotic cells (from 4.2% to 65.6% for U87 cells, p < 0.01; from 5.7% to 62.8% for U251 cells, p < 0.01) was dramatically increased in a dose-dependent manner after Sch B treatment compared with the control group. Furthermore, morphological changes in the apoptotic cells were investigated using Hoechst 33342 staining. When U87 and U251 cells were exposed to different doses of Sch B for 48 h, the number of apoptotic cells containing condensed and fragmented nuclei increased significantly as the dose of Sch B increased (Figure 4C,D). These results indicate that the apoptotic pathway plays a pivotal role in the anti-proliferation effect of Sch B on U87 and U251 cells.

### Sch B decreases mitochondrial membrane potential (ΔΨm) in glioma cells

**Figure 5:**
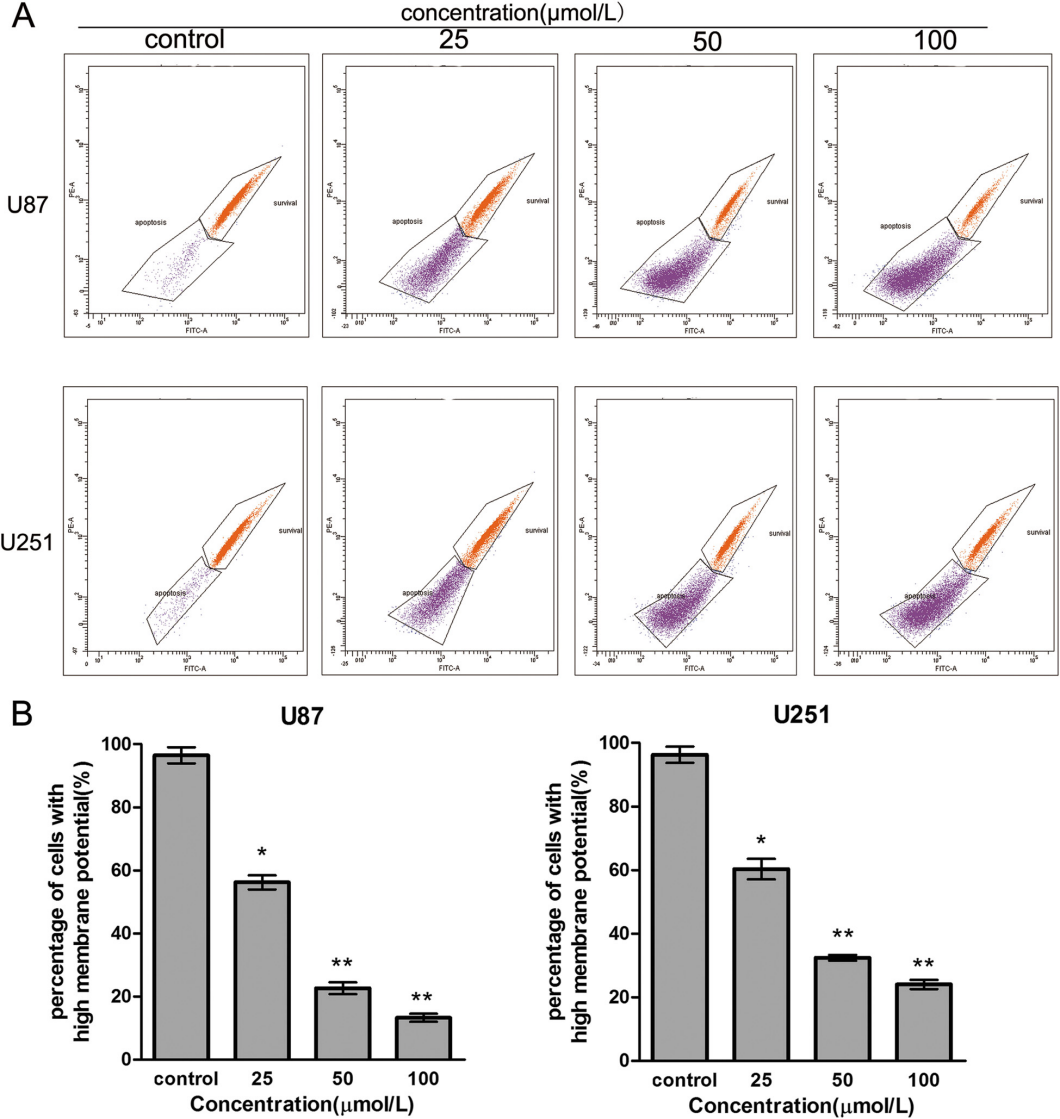
**Sch B decreases the mitochondrial membrane potential (ΔΨm) in glioma cells.** U87 and U251 cells were treated with Sch B (0, 25, 50, 100 μmol/L) for 48 h. **A**. Flow cytometric analysis of ΔΨm by rhodamine 123 staining. **B**. The corresponding histogram shows the percentages of cells with high ΔΨm (survival) and low ΔΨm (apoptosis). Values represent the means ± SD (n = 3). *P < 0.05; **P < 0.01; ***P < 0.001 compared with the control group.

Mitochondria are key regulators of apoptosis, and apoptosis mediated by the mitochondrial pathway is often associated with decreased ΔΨm. To examine whether the mitochondrial membrane integrity is damaged by Sch B treatment, rhodamine 123 was used to determine ΔΨm in Sch-B treated glioma cells. Compared with the control cells, Sch B treatment induced a dose-dependent decrease in ΔΨm (Figure 5A,B). We found that more than 80% of U87 cells and 76% of U251 cells showed a reduction in ΔΨm after treatment with 100 μmol/L Sch B for 48 h. These findings suggest that Sch B could reduce the mitochondrial membrane potential and induce mitochondrial dysfunction in glioma cells, indicating that Sch B promotes glioma cell apoptosis through a mitochondrial-dependent apoptotic pathway.

### Sch B-induced apoptosis via the regulation of caspase-3 and Bcl-2 family members in glioma cells

**Figure 6:**
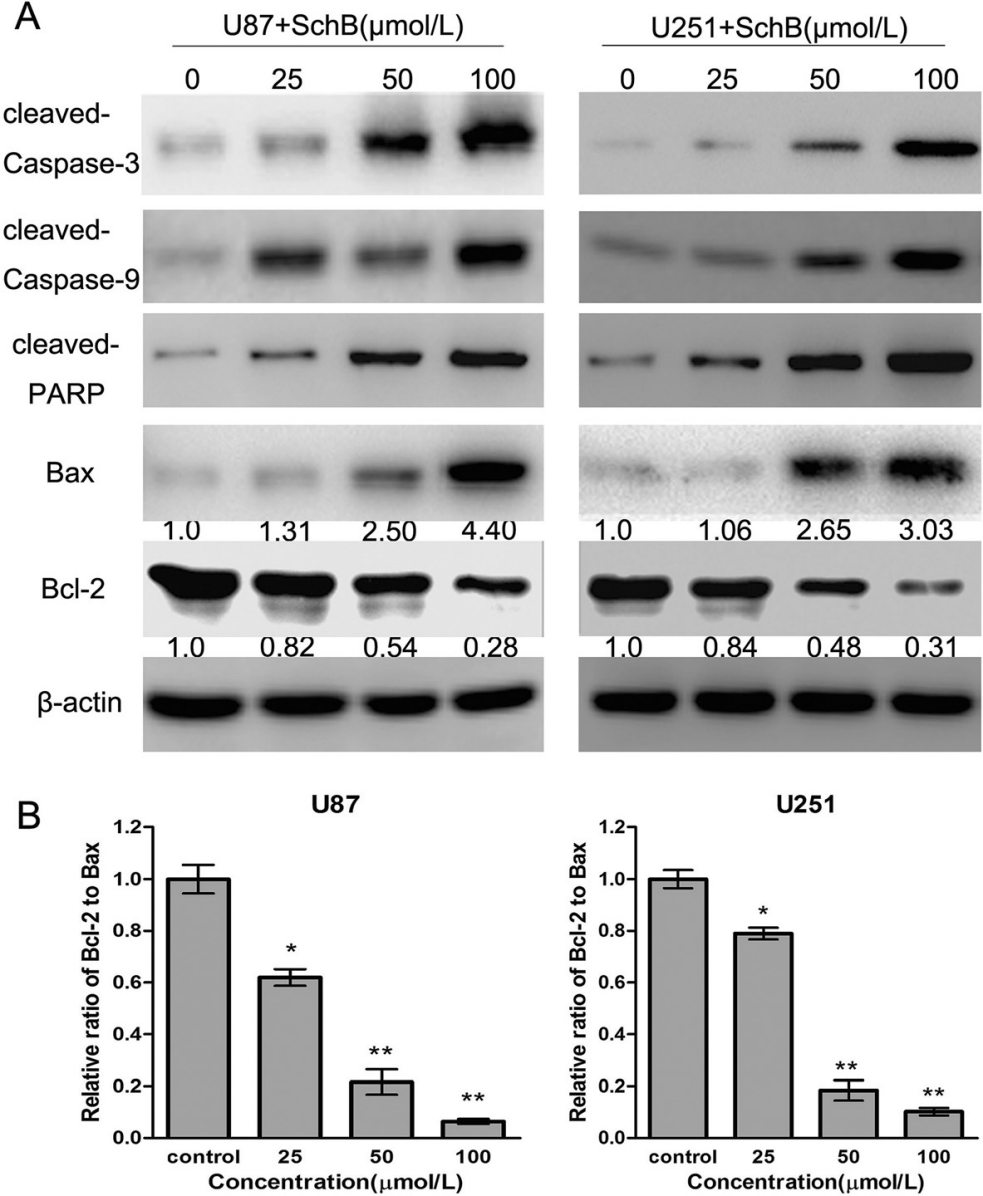
**Sch B induces apoptosis via the regulation of apoptosis-related proteins in glioma cells. A**. After treatment with Sch B (0, 25, 50, 100 μmol/L) for 48 h, cell lysates of U87 and U251 cells were prepared, and the expression of cleaved caspase-3, cleaved PARP, Bax and Bcl-2 were analysed using western blot. β-actin was used as a loading control. **B**. The ratio of Bcl-2 to Bax was calculated by band density. The results were shown as the mean ± SD compared with the control (designated as 1.00). *P < 0.05; **P < 0.01 compared with the control group.

Many studies have shown that the Caspase family, Bcl-2 family and PARP play critical roles in apoptosis [16-18]. To investigate the potential molecular mechanisms responsible for Sch B-induced apoptosis of U87 and U251 cells, we detected the expression of apoptosis-related proteins (Bax, Bcl-2, cleaved caspase-3 and cleaved caspase-9) after treating the cells with various doses of Sch B for 48 h. As shown in Figure 6A, Sch B treatment leads to the upregulation of cleaved PARP, Bax, cleaved caspase-9, and cleaved caspase-3 and the downregulation of Bcl-2 in a dose-dependent manner. In addition, the Bcl-2 (antiapoptotic) to Bax (proapoptotic) ratio was significantly reduced compared with the control group (Figure 6B). In summary, Sch B promotes apoptosis through the regulation of apoptosis-related protein expression in glioma cells.

### Sch B reduced tumour growth in vivo

**Figure 7:**
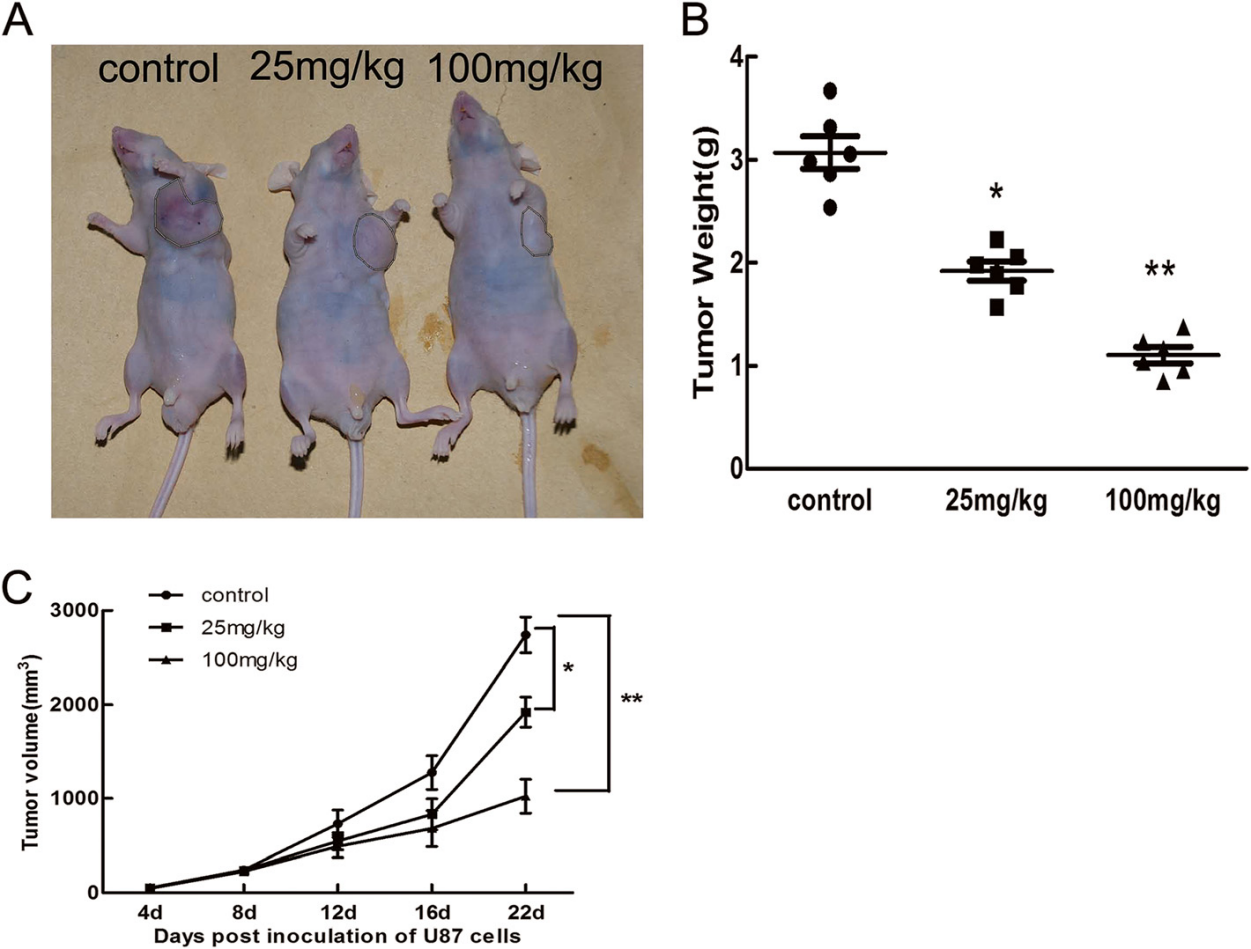
**Sch B inhibited the growth of U87 cells** ***in vivo*** **. A**. U87 cells were subcutaneously injected into the left flank of nude mice. Then, 0.1 mL vehicle (PBS) or Sch B (25 and 100 mg/kg) was intraperitoneally administered to the animals every 2 days for up to 20 days. Photos of one representative mouse (n = 6) from each group are shown. **B**. Tumours were excised and weighed in each group. *P < 0.05, **P < 0.01; ***P < 0.001 vs. the control group. **C**. Tumor dimensions were periodically measured using callipers.

To further investigate the effect of Sch B on tumour growth *in vivo*, we intraperitoneally injected vehicle (PBS) or Sch B (25 mg/kg, 100 mg/kg) into nude mice carrying subcutaneous U87 tumour xenografts every 2 days for up to 20 days. As shown in Figure 7B, the tumor weight was dramatically reduced after treatment with Sch B(1.98 ± 0.07g in the 25 mg/kg group *vs.* 3.08 ± 0.15 in the control group, p < 0.05; 1.08 ± 0.08g in the 100mg/kg group *vs.* 3.08 ± 0.15 in the control group, p < 0.01). Our results showed that Sch B significantly inhibited tumour growth in a dose-dependent manner compared with tumours in the control group (Figure 7A,B,C).

## Discussion

It has been reported that Sch B possesses anti-neoplastic ability in a broad range of human cancers by promoting apoptosis [10-12]. However, the effect of Sch B on glioma cells and its underlying mechanisms remain largely unknown. In the present study, we demonstrated that Sch B inhibited proliferation, induced apoptosis and G0/G1 phase arrest in glioma cells and strongly restrained the tumour growth *in vivo.* Thus, Sch B may be a promising drug to treat gliomas.

Results of cell cycle distribution experiment in the presence of Sch B demonstrated that it induced G0/G1 phase arrest after 48 h of treatment and that prompted us to further investigate the mechanism of cell cycle arrest. Cyclins and CDKs play key roles in cell cycle regulation [19]. Cyclin D1, one of the key cell cycle regulatory molecules [20,21], can form a complex with CDK4 to promote the transition of cells from G0/G1 to S phase by catalysing the phosphorylation of retinoblastoma (Rb) [22-24]. Indeed, we observed that Sch B downregulated the expression of cyclin D1 and CDK4 in both U87 and U251 cells (Figure 3C). Based on these data, we believe that Sch B arrests the cell cycle in G0/G1-phase to suppress the proliferation of glioma cells by reducing the expression of cyclin D1 and CDK4.

The deregulation of apoptosis has frequently been observed in various cancers; thus, the induction of apoptosis has been proposed as an important strategy to treat cancer [25,26]. Generally, there are two major pathways that control the initiation of apoptosis: the death-receptor-induced extrinsic pathway and the mitochondria-apoptosome-mediated intrinsic pathway, both of which ultimately activate a series of effector caspases and apoptosis effector molecules [27]. In the extrinsic pathway, the binding of ligands to membrane death-receptors leads to the activation of caspase-8; In the intrinsic pathway, Bax induces the release of cytochrome c from the mitochondria, leading to the apoptosome formation and activation of caspase 9, and subsequent caspase 3 activation. Caspase-3 is one of the most important executioner caspases and is capable of cleaving many important cellular substrates, such as PARP, resulting in apoptosis progression, morphological changes, and DNA fragmentation [28]. In our study, Sch B treatment of U87 and U251 cells activated caspase-9 and caspase-3, accompanied by increased PARP cleavage, suggesting that the mitochondrial pathway was involved in Sch B-induced apoptosis. Proteins in the Bcl-2 family, including the apoptosis-promoting protein Bax and anti-apoptotic protein Bcl-2, are the key regulators of the mitochondria-mediated apoptosis pathway [29,30]. It has been reported that a low Bcl-2 to Bax ratio can cause ΔΨm collapse, the release of cytochrome c, and subsequent apoptosis [31]. In the present study, we found that the ratio of Bcl-2 to Bax was reduced by 15.6- and 10.24-fold after treatment with 100 μmol/L Sch B in U87 and U251 cells, respectively, compared with that of the control group. As a result, a significant decrease of ΔΨm was observed in giloma cells.

To further address the antitumor activity of Sch B against glioma, the animal work should be taken entirely. Due to the prolonged tissue culture, the genetic and morphologic characteristics of established cell lines often differ from the primary human tumors. To overcome these shortcomings, Sarkaria lab [32] have developed a model of primary glioblastoma multiforme (GBM) xenografts that were established by engraftment of patient tumor specimens directly into the flank of nude mice. These xenograft lines remains key genetic features of the corresponding patient tumor, this is highly useful for both basic and translational studies. We can use this model to test the effect of Sch B on patient tumor specimens. The extension of animal numbers is also needed to further confirm the clinical significance of Sch B on the treatment of glioma.

## Conclusion

In summary, our study suggests that Sch B attenuates the proliferation of U87 and U251 cells and arrests cell cycle in the G0/G1 phase. Sch B induces cell apoptosis by increasing Bax, cleaved caspase-9, cleaved caspase-3, and cleaved PARP and reducing Bcl-2 protein expression in glioma cells. Furthermore, Sch B dramatically inhibited tumour growth *in vivo*. All of the above results indicate that Sch B may be a promising drug for glioma treatment. However, further studies are needed to understand the fundamental mechanism underlying the apoptosis and cell cycle arrest induced by Sch B in glioma cells.

## Methods

### Drugs and antibodies

Sch B was purchased from Sigma-Aldrich (St. Louis, MO, USA) and dissolved in dimethyl sulfoxide (DMSO, Sigma-Aldrich) to obtain a 100 mM stock solution, which was then diluted in the culture medium to yield the desired Sch B concentration. An equal volume of DMSO in complete culture medium was used as the vehicle control. To eliminate the cytotoxicity of DMSO, the final concentration of DMSO for all experiments was maintained at less than 0.1%. Rhodamine 123 and 3-[4,5-dimethylthiazol-2-yl]-2,5-diphenyl-tetrazolium bromide (MTT) were purchased from Sigma Chemical Co. Primary antibodies against Bcl-2, Bax, cleaved caspase-9, cleaved PARP, cleaved caspase-3, cdc-2, cyclin D1, and GAPDH and secondary antibodies (goat anti-rabbit) were all purchased from Cell Signaling Technology (Danvers, MA, USA).

### Cell lines and culture

The human glioma U87 and U251 cell lines were purchased from the Shanghai Institute of Cell Biology, Chinese Academy of Sciences (CAS, Shanghai, China). All cells were cultured in high-glucose DMEM (Gibco, Grand Island, NY, USA) supplemented with 10% foetal bovine serum (Gibco), 100 μg/mL streptomycin, and 100 U/mL penicillin (HyClone, USA).The cells were cultured in an atmosphere containing 5% CO_2_ at 37°C.

### Cell viability assay

The viability of cells treated with Sch B was measured as previously described [33]. Briefly, cells were seeded into 96-well culture plates at 5,000 cells/well, incubated overnight, then treated with 0, 20, 40, 60, 80 and 160 μmol/L Sch B for 24, 48 and 72 h. Next, added 20 μL MTT solution (5 mg/mL) to each well for another 4 h after treatment. In order to solubilise the formazan crystals, 100 μL DMSO was added to each well. The absorbance was determined at 490 nm using a microplate reader (Bio-Tek, Winooski, VT, USA). The cell survival rate was measured as the absorbance compared with that of untreated controls. The results represent the average of 3 independent experiments.

### Colony formation assay

Briefly, cells in the logarithmic growth phase were seeded into 6-well plates (Corning, Corning, NY, USA) at a density of 400 cells/well and allowed to grow for 24 h. The cells were then treated with Sch B (0, 4, 8 and 16 μmol/L) for another 48 h. The Sch B-containing medium was then discarded, and the cells were washed with PBS and cultured to form colonies in complete medium for another 14 days. Next, the cells were fixed with 4% paraformaldehyde for 15 min at room temperature and stained with 0.1% crystal violet (Sigma-Aldrich) for 30 min. After washing, the total number of colonies (>50 cells/colony) was counted manually and the stained colonies were photographed using a microscope (Leica, Wetzlar, Germany). The results were expressed as the average of three independent experiments.

### Cell cycle analysis

U87 and U251 cells were treated with various concentrations of Sch B for 48 h. Both adherent and floating cells were harvested, washed twice in cold PBS, and fixed in 70% ethanol at 4°C overnight. After fixation, the cells were washed and incubated in staining buffer (10 mg/mL RNase and 1 mg/mL propidium iodide (Sigma-Aldrich)) at 37°C in the dark for 30 min. Subsequently, the samples were analysed using flow cytometry (BD Biosciences, San Diego, CA, USA). The percentages of cells in the G0/G1, S, and G2/M phases were calculated using Cell Quest acquisition software (BD Biosciences).

### Cell apoptosis assay

Apoptosis was analysed using the annexin V/PI apoptosis kit according to the manufacturer’s recommendations (BD Biosciences, San Diego, CA, USA). Briefly, Two hundred thousand cells (2 × 10^5^) were first seeded into 6-well plates and treated with Sch B (0, 25, 50, and 100 μmol/L) for 48 h. The cells were then harvested and resuspended at a density of 1 × 10^6^ cells/mL in binding buffer. Then 5 μL annexin V-FITC and 5 μL PI (100 μg/mL) were added to the suspension cells. After incubation for 15 min in the dark at room temperature, 400 μL of the binding buffer was added to the cell suspension. The stained cells were then immediately analysed using flow cytometry (BD Biosciences, San Diego, CA, USA). Each sample was assayed in duplicate, and the experiment was repeated three times.

### Hoechst 33342 staining assay

Nuclear fragmentation was determined by staining apoptotic nuclei with Hoechst 33342. U87 and U251 cells were treated with various concentrations of Sch B for 48 h, the cells were harvested, washed in cold PBS and fixed with acetic acid:ethanol (1:3) for 15 min at room temperature. The fixed cells were then washed and stained with 5 μg/mL Hoechst 33342 for 10 min. Digital images were captured under a fluorescence microscope (Leica, Germany).

### Mitochondrial membrane potential (ΔΨm) assay

Rhodamine 123 (Rho123) was used to determine the mitochondrial membrane potential (ΔΨm) as previously described [34]. Briefly, after treatment with various concentrations of Sch B for 48 h, U87 and U251 cells were collected and washed twice with cold PBS. The cells were then stained with Rhodamine 123 (Sigma-Aldrich) for 30 min at 5% CO_2_ and 37°C in the dark. The samples were then analysed using flow cytometry (BD Biosciences, San Diego, CA, USA). Each assay was carried out in triplicate, and the results were expressed as the mean ± SD.

### Western blot analysis

U87 and U251 cells were treated with Sch B (0, 25, 50 and 100 μmol/L) for 48 h, total protein was extracted using RIPA buffer (Beyotime Institute of Biotechnology, Beijing, China) supplemented with protease inhibitor (Roche Applied Science, Indianapolis, IN, USA) at 4°C for 5 min. The protein concentration was determined using the bicinchoninic acid (BCA) assay kit (Beyotime). Total protein samples (40 μg/lane) were separated using 10% SDS-PAGE and then electrophoretically transferred to polyvinylidene difluoride membranes (Millipore, Bedford, MA, USA). After blocking with 5% skim milk for 1–2 h at room temperature, the membranes were then incubated with the indicated primary antibodies against Bcl-2, Bax, cleaved PARP, cleaved caspase-9, cleaved caspase-3, CDK4, cyclin D1, and β-actin (1:1000) at 4°C overnight. The membranes were then washed with TBST buffer for 3 × 5 minutes and incubated with the secondary antibodies (HRP-conjugated goat anti-rabbit IgG) for 1 h at room temperature. The bands were detected using enhanced chemiluminescence and visualised by a Gel Doc 2000 (BioRad, Hercules, CA, USA).

### In vivo tumour xenograft study

BALB/c homozygous (nu/nu) nude mice (6–8 weeks old, 18–20 g body weight) that had been bred in house were maintained in a specific pathogen-free environment. U87 cells in log-phase growth (2.5 × 10^6^) were suspended in 100 μL PBS and then subcutaneously injected into the left flank of the nude mice. Twenty-four hours post inoculation, the mice were randomly divided into three groups (6 mice/group). The control group was administered PBS intraperitoneally and the other two treatment groups were administered Sch B (25 and 100 mg/kg) intraperitoneally in a volume of 0.2 mL every 2 days for up to 20 days. On day 22, all mice were sacrificed, and the tumours were excised and weighed. The experimental procedure was conducted according to the institutional animal care guideline and approved by the Laboratory Animal Ethics Committee of Wenzhou Medical University with the reference number wydw2014-0026.

### Statistical analysis

All data were expressed as the mean ± SD of at least three independent experiments. Differences between groups were analyzed using the Student’s *t* test,. A P-value of less than 0.05 (denoted by *) was considered to be statistically significant.
